# Improving data splitting for classification applications in spectrochemical analyses employing a random-mutation Kennard-Stone algorithm approach

**DOI:** 10.1093/bioinformatics/btz421

**Published:** 2019-05-22

**Authors:** Camilo L M Morais, Marfran C D Santos, Kássio M G Lima, Francis L Martin

**Affiliations:** 1 School of Pharmacy and Biomedical Sciences, University of Central Lancashire, Preston PR1 2HE, UK; 2 Institute of Chemistry, Biological Chemistry and Chemometrics, Federal University of Rio Grande do Norte, Natal 59072-970, Brazil

## Abstract

**Motivation:**

Data splitting is a fundamental step for building classification models with spectral data, especially in biomedical applications. This approach is performed following pre-processing and prior to model construction, and consists of dividing the samples into at least training and test sets; herein, the training set is used for model construction and the test set for model validation. Some of the most-used methodologies for data splitting are the random selection (RS) and the Kennard-Stone (KS) algorithms; here, the former works based on a random splitting process and the latter is based on the calculation of the Euclidian distance between the samples. We propose an algorithm called the Morais-Lima-Martin (MLM) algorithm, as an alternative method to improve data splitting in classification models. MLM is a modification of KS algorithm by adding a random-mutation factor.

**Results:**

RS, KS and MLM performance are compared in simulated and six real-world biospectroscopic applications using principal component analysis linear discriminant analysis (PCA-LDA). MLM generated a better predictive performance in comparison with RS and KS algorithms, in particular regarding sensitivity and specificity values. Classification is found to be more well-equilibrated using MLM. RS showed the poorest predictive response, followed by KS which showed good accuracy towards prediction, but relatively unbalanced sensitivities and specificities. These findings demonstrate the potential of this new MLM algorithm as a sample selection method for classification applications in comparison with other regular methods often applied in this type of data.

**Availability and implementation:**

MLM algorithm is freely available for MATLAB at https://doi.org/10.6084/m9.figshare.7393517.v1.

## 1 Introduction

Data splitting is a process used to separate a given dataset into at least two subsets called ‘training’ (or ‘calibration’) and ‘test’ (or ‘prediction’). This step is usually implemented after pre-processing, when the samples’ spectra have been corrected for noise or undesired variability. These subsets are used towards constructing chemometric models for quantification or classification applications. In quantification, calibration models are built to assign a concentration or discrete value to a sample based on its spectral signature, whilst in classification applications, samples or experimental observations are assigned to ‘classes’ based on their spectrochemical signature. This is made by using chemometric methods such as principal component analysis linear discriminant analysis (PCA-LDA) (Morais and Lima, 2018), partial least squares discriminant analysis (PLS-DA) (Brereton and Lloyd, 2014), or support vector machines (SVM) (Cortes and Vapnik, 1995). Sometimes, especially for large datasets, an extra subset called ‘validation’ is also obtained, containing measurements observations used for optimizing factors in the chemometric model, such as the number of principal component (PCs) in PCA-LDA, latent variables in PLS-DA and kernel parameters in SVM. When the validation set is not present, cross-validation is applied. In this case, samples from the training set are used in an iterative validation process for optimizing these models parameters. This is made by firstly removing a certain number of samples from the training set and then building the classification model with the remaining samples, where the removed samples are predicted as a temporary validation set. This is performed for a certain number of repetitions until all training samples are excluded once from the training set and predicted as a temporary validation set. One of the most popular cross-validation methods is the leave-one-out cross-validation, where only one sample is removed from the training set per each iteration. A misclassification error is then calculated for this temporary validation set, where different models parameters, such as different number of factors or principal components, are tested. The training model with the lowest cross-validation error is then chosen as final, where the classification parameters that led to the lowest cross-validation error value are selected. The samples primarily excluded from modelling (test set) are used for final model evaluation, since they are considered as being external to the model (blind). In this case, one simulates how the model would behave in the presence of new observations, though they are often measured in the same experiment with the training samples.

To avoid the presence of bias introduced by manual data splitting, there are a number of computational methods that can be used for sample selection, such as based on leverage (Wang *et al.*, 1991), random selection (RS) or Kennard-Stone (KS) algorithm (Kennard and Stone, 1969). RS and KS are the most used methods for sample selection; the former due to its simplicity and the latter due to its adaptation to analytical chemistry applications, since it allows a training model covering most sources of variations within the dataset, ensuring the training model is more representative of the whole dataset. Currently, the original KS paper (Kennard and Stone, 1969) has >1000 citations, being the method of choice in many classification applications.

Although including as much variability as possible within the training model provides a good predictive performance, sometimes random phenomena might occur with new samples in a test set, in particular when samples come from complex matrices. An example of this is biological-derived samples. Biological samples can be affected by a series of factors that are difficult to include in relatively small datasets. For example, in clinical applications the spectrochemical response of a ‘healthy’ and ‘disease’ sample may vary according to changes in diet and lifestyle (Lindon *et al.*, 2017). The same applies for bacteria or viruses extracted from certain media, since environmental variations may also change their spectral signature. Additionally, random factors such as genetic mutations might affect the predictive performance of a classification model for biological samples in the future. These phenomena add a degree of ‘randomness’ in the predictive behaviour of a classifier, since more extrapolations might be needed to address all of these issues. Thus, having in mind the inclusion of as much representativeness as possible in the training model but with a small degree of randomness, we propose a new algorithm based on a random-mutation Kennard-Stone approach; we call this the Morais-Lima-Martin (MLM) algorithm.

Towards comparison of the predictive response of MLM with RS and KS, we tested classification models on six real-world spectrochemical datasets using PCA-LDA, where the predictive performance in terms of accuracy, sensitivity and specificity were evaluated. In addition, simulations with normally distributed randomly data were performed to evidence the performance of the MLM algorithm in comparison with the RS and KS method.

## 2 Materials and methods

**Datasets.** Six real-world datasets were used towards comparing the classification performance of RS, KS and MLM algorithms. Dataset 1 contains 280 infrared (IR) spectra of two Cryptococcus fungi specimens acquired via attenuated total reflection Fourier-transform infrared (ATR-FTIR) spectroscopy. This dataset is publically available at https://doi.org/10.6084/m9.figshare.7427927.v1. Class 1 is composed of 140 spectra of *Cryptococcus neoformans* samples and class 2 of 140 spectra of *Cryptococcus gattii* samples. Spectra were acquired in the 400–4000 cm^−1^ spectral range with a resolution of 4 cm^−1^ and 16 co-added scans using a Bruker VERTEX 70 FTIR spectrometer (Bruker Optics, Ltd., UK). The spectral data were pre-processed by excising the biofingerprint region (900–1800 cm^−1^), which was followed by automatic weighted least squares (AWLS) baseline correction and normalization to the Amide I peak (1650 cm^−1^). More details regarding this dataset can be found in literature (Costa *et al.*, 2016; Morais *et al.*, 2017).

Dataset 2 contains 240 IR spectra derived from formalin-fixed paraffin-embedded brain tissues separated into two classes. Class 1 contains 140 spectra from normal brain tissue, and class 2 contains 100 spectra from glioblastoma brain tissue. Spectra were collected via ATR-FTIR spectroscopy using a Bruker VECTOR 27 FTIR spectrometer with a Helios ATR attachment (Bruker Optics, Ltd., UK). The raw spectra, acquired in the 400–4000 cm^−1^ spectral range with a resolution of 8 cm^−1^ and 32 co-added scans, were pre-processed by excising the biofingerprint region (900–1800 cm^−1^), which was followed by rubberband baseline correction and normalization to the Amide I peak (1650 cm^−1^). This dataset is publicly available as part of the IRootLab toolbox (http://trevisanj.github.io/irootlab/) (Trevisan *et al.*, 2013), and more information about it can be found in Gajjar *et al.* (2012).

Dataset 3 contains 183 IR spectra distributed into 3 classes. Class 1 contains 59 spectra of Syrian hamster embryo (SHE) cells treated with benzo[a]pyrene (B[a]P), class 2 contains 62 spectra of SHE cells treated with 3-methylcholanthrene (3-MCA) and class 3 contains 62 spectra of SHE cells treated with anthracene (Ant). Spectra were acquired in the 400–4000 cm^−1^ spectral range with a resolution of 8 cm^−1^ by using a Bruker TENSOR 27 spectrometer with a Helios ATR attachment (Bruker Optics, Ltd., UK). Pre-processing was performed by excising the biofingerprint region (900–1800 cm^−1^), which was followed by rubberband baseline correction and normalization to the Amide I peak (1650 cm^−1^). This dataset is publicly available as part of the IRootLab toolbox (http://trevisanj.github.io/irootlab/) (Trevisan *et al.*, 2013), and further information can be found in Trevisan *et al.* (2010).

Dataset 4 contains 270 IR spectra from blood samples divided into four classes. Class 1 is composed of 90 IR spectra of control samples, class 2 contains 88 spectra from patients with Dengue, class 3 contains 66 spectra from patients with Zika and class 4 contains 26 spectra from patients with Chikungunya. This dataset is publically available at https://doi.org/10.6084/m9.figshare.7427933.v1. Spectra were collected in ATR mode by using a Bruker VERTEX 70 FTIR spectrometer (Bruker Optics, Ltd., UK). Acquisition was performed in the 400–4000 cm^−1^ spectral range with a resolution of 4 cm^−1^ and 16 co-added scans. Pre-processing was performed by excising the biofingerprint region (900–1800 cm^−1^), which was followed by Savitzky-Golay smoothing (window of 7 points) (Savitzky and Golay, 1964), AWLS baseline correction and normalization to the Amide I peak (1650 cm^−1^). Further details about this dataset can be found in Santos *et al.* (2018).

Dataset 5 contains 351 Raman spectra of blood plasma divided into two classes: 162 spectra of healthy individuals (class 1), and 189 spectra of ovarian cancer patients (class 2). This dataset is publicly available at https://doi.org/10.6084/m9.figshare.6744206.v1. Raman spectra were collected using an InVia Renishaw Raman spectrometer coupled with a charge-coupled device (CCD) detector and Leica microscope, with 5% laser power (785 nm), 5x objective magnification, 10 s exposure time and 2 accumulations in the spectral range of 400–2000 cm^−1^. The spectral data were pre-processed by Savitzky-Golay smoothing (window of 15 points), AWLS baseline correction and vector normalization. Further details about this dataset can be found in Paraskevaidi *et al.* (2018).

Dataset 6 contains 322 surface-enhanced Raman spectroscopy (SERS) spectra of blood plasma also divided into two classes: 133 spectra of healthy individuals (class 1), and 189 spectra of ovarian cancer patients (class 2). This dataset is publicly available at https://doi.org/10.6084/m9.figshare.6744206.v1. SERS spectra were collected using the same settings for dataset 5 but, in this case, silver nanoparticles were mixed with the biofluid before spectral acquisition. The spectral pre-processing was performed using Savitzky-Golay smoothing (window of 15 points), AWLS baseline correction and vector normalization. Further details about this dataset can be found in Paraskevaidi *et al.* (2018).

Simulations were also performed with simulated data. This data were generated for each simulation (1000 simulations) based on a normally distributed random matrix with size of 100 × 1000 for class 1, and 100 × 1000 for class 2 (100 observations, 1000 variables per observation). The matrix values ranged randomly from -10 to 10 units. A shift of 5 units was randomly added to class 2 to create a difference between the classes. The codes to produce class 1 and class 2 in MATLAB are ‘class_1 = randn(100, 1000).*randn(100, 1000);’ and ‘class_2 = (randn(100, 1000)+5).*randn(100, 1000);’. Class 1 and class 2 were generated for each simulation (1000 times), where all algorithms (RS, KS and MLM) were independently applied per each simulation.

**Software.** Data analysis was performed within the MATLAB R2014b (MathWorks, Inc., USA) environment. Pre-processing was performed using PLS Toolbox 7.9.3. (Eigenvector Research, Inc., USA) and classification was performed using the Classification Toolbox for MATLAB (http://www.michem.unimib.it/) (Ballabio and Consonni, 2013). RS, KS and MLM algorithms were performed using laboratory-generated routines. MLM algorithm is public available at https://doi.org/10.6084/m9.figshare.7393517.v1.

**Fig. 1.**
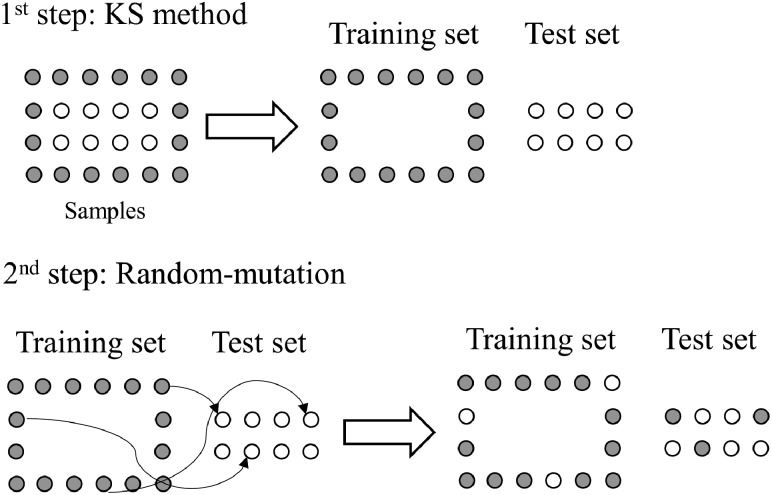
Illustration of the MLM algorithm based on a random-mutation of the Kennard-Stone (KS) method. Adapted from Morais *et al.* (2018)

**Sample selection.** Samples were divided into training (70%) and test (30%) sets using, independently, the RS, KS or MLM algorithms. RS is based on a random sample selection where spectra from the original dataset are randomly assigned to training or test. KS algorithm is based on an Euclidian distance calculation, where the sample with maximum distance to all other samples are selected, then the samples which are as far away as possible from the selected samples are selected, until the selected number of samples is reached. This means that the samples are selected in such a way that they will uniformly cover the complete sample space, reducing the need for extrapolation of the remaining samples. MLM algorithm, based on a KS-based approach, applies a KS method to the data, as described before; then, a random-mutation factor is used in the KS results, where some samples from the training set are transferred to the test set, and some samples from the test set are transferred to training. Herein, the mutation factor was set at 10%. This value is inspired in the mutation probability of genetic algorithms (Morais *et al.*, 2019), where 10% is a common threshold employed to keep a balance between the degree of randomness and model convergence. MLM algorithm is visually illustrated in Figure 1.

**Classification.** Classification was performed based on a PCA-LDA algorithm. For this, initially a principal component analysis (PCA) model is applied to the pre-processed data, decomposing the spectral space into a small number of PCs representing most of the original data-explained variance (Bro and Smilde, 2014). Each PC is composed of scores and loadings, the former representing the variance on samples direction, and the latter the variance on variables (e.g. wavenumber) direction. Then, the PCA scores are used as input for a linear discriminant analysis (LDA) classifier. LDA performs a Mahalanobis distance calculation to linearly classify the input space (PCA scores) into at least two classes (Dixon and Brereton, 2009; Morais and Lima, 2018). The LDA classification scores ($L_{\mathrm{ik}}$) can be calculated in a non-Bayesian form as (Dixon and Brereton, 2009; Morais and Lima, 2018):

(1) $$L_{\mathrm{ik}}={\left(x_{i}-{\bar{x}}_{k}\right)}^{T}C_{\mathrm{pooled}}^{-1}\left(x_{i}-{\bar{x}}_{k}\right)$$

where $x_{i}$ is a vector containing the input variables for sample i; ${\bar{x}}_{k}$ is the mean vector of class k; $C_{\mathrm{pooled}}$ is the pooled covariance matrix between the classes; and, T represents the matrix transpose operation. Model optimization was performed using cross-validation venetian blinds with 10 splits.

The PCA-LDA classification performance was evaluated by means of accuracy, sensitivity and specificity calculations. Accuracy represents the total number of samples correctly classified considering true and false negatives; sensitivity measures the proportion of positives that are correctly identified; and, specificity measures the proportion of negatives that are correctly identified (Morais and Lima, 2017). These parameters are calculated as follows:

(2) Accuracy (%)=((TP+TN)/(TP+FP+TN+FN))×100

(3) Sensitivity (%)=(TP/(TP+FN))×100

(4) Specificity (%)=(TN/(TN+FP))×100

where TP stands for true positives; TN for true negatives; FP for false positives; and, FN for false negatives.

## 3 Results

**Fig. 2.**
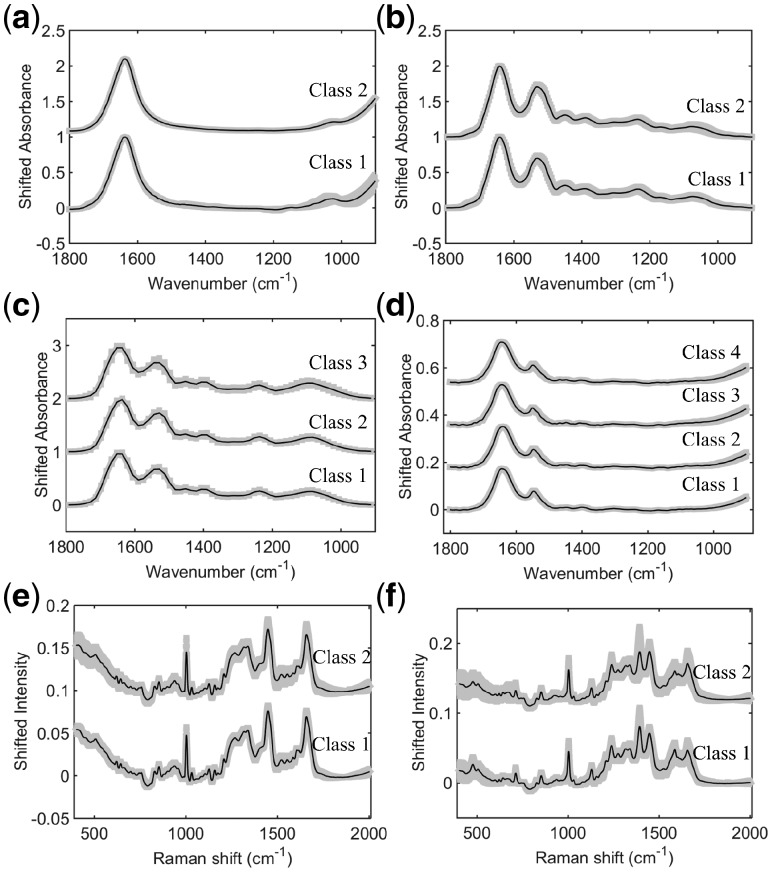
Mean pre-processed spectrum with standard deviation (shaded) for each class in dataset 1 (**a**), 2 (**b**), 3 (**c**), 4 (**d**), 5 (**e**) and 6 (**f**)

Six real-world datasets were evaluated using different data splitting techniques: RS, KS and our new MLM algorithm. These datasets are composed of IR and Raman spectra from biological-derived applications involving: IR spectra of fungi (dataset 1); IR spectra of cancer brain tissue (dataset 2); IR spectra for toxicological study (dataset 3); IR spectra of viruses (dataset 4); Raman spectra of plasma for ovarian cancer detection (dataset 5); and, SERS spectra of plasma for ovarian cancer detection (dataset 6). Figure 2 shows the pre-processed mean spectrum with standard deviation for each class in datasets 1–6. The pre-processed spectra from these datasets were used as input for the sample selection techniques, where their classification performances were evaluated via the PCA-LDA algorithm.

**Fig. 3.**
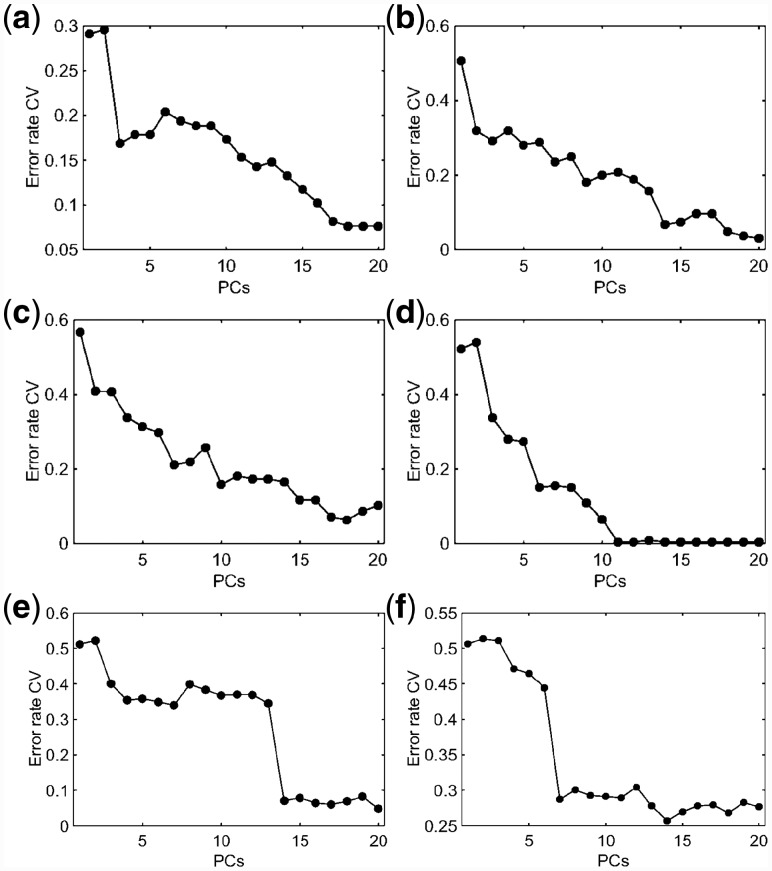
PCA-LDA cross-validation error rate for datasets 1 (**a**), 2 (**b**), 3 (**c**), 4 (**d**), 5 (**e**) and 6 (**f**). CV: cross-validation; PCs: principal components

Dataset 1 is composed of 280 IR spectra for two fungi specimens groups (*Cryptococcus neoformans* [class 1]; *Cryptococcus gattii* [class 2]), each class having 170 spectra each. Both fungi classes are pathogenic agents responsible for causing Cryptococcosis in humans, differing in their epidemiology, host range, virulence, antifungal susceptibility and geographic distribution (Morais *et al.*, 2017). From a clinical point of view, Cryptococcus neoformans is a pathogen with a tendency to attack the central nervous system and its effects are mainly noted in immunosuppressed patients, whereas *Cryptococcus gattii* targets the lungs of immunocompetent, healthy individuals (Morais *et al.*, 2017). RS, KS and MLM were independently applied to the pre-processed spectra separating 70% of them for training and 30% for testing. Cross-validated PCA-LDA was applied for model construction using three PCs (99% cumulative explained variance) selected according to the minimum cross-validation error rate within the minimum number of PCs (Fig. 3). The model fitting performance is shown in Table 1, where the best training (84%) and cross-validation (83%) accuracy are observed using RS algorithm. KS generates the worst fitting performance with 80% accuracy in both training and cross-validation. The MLM algorithm shows an intermediary performance with 83% and 82% accuracy in training and cross-validation, respectively.

**Table 1.** PCA-LDA fitting accuracy for training and cross-validation (CV) varying with the sample selection method (RS: random selection; KS: Kennard-Stone; MLM: Morais-Lima-Martin) applied in datasets 1–6

Dataset	Sample selection method	Training accuracy (%)	CV accuracy (%)
1	RS	84	83
	KS	80	80
	MLM	83	82
2	RS	85	83
	KS	81	80
	MLM	82	77
3	RS	86	84
	KS	83	82
	MLM	84	80
4	RS	92	91
	KS	90	90
	MLM	93	90
5	RS	93	93
	KS	89	88
	MLM	91	88
6	RS	74	72
	KS	75	72
	MLM	76	75

**Fig. 4.**
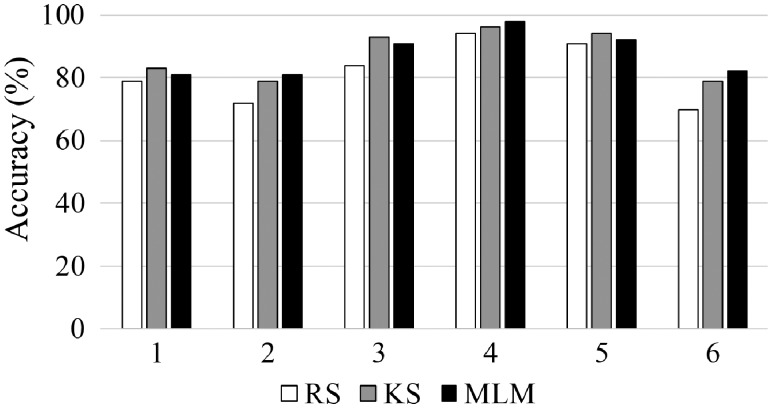
Accuracy in the test set obtained by PCA-LDA varying with the sample selection method (RS: random selection; KS: Kennard-Stone; MLM: Morais-Lima-Martin) applied in datasets 1–6

Although the best fitting accuracy, the RS-based model exhibits a very poor sensitivity, at 69%, in the test set (Table 2). The specificity is high (88%), but the model seems to have a poor balance in terms of sensitivity and specificity, indicating that one class is much better classified than the other. The KS-based model with the worst fitting gives the best specificity (98%), but the sensitivity remains the same. On the other hand, the MLM-based model shows the best well-balanced performance, where the specificity falls to 78%, but the sensitivity increases to 74%, indicating that both classes are well-classified, and the model is not skewed towards a good classification of just one of the classes. Overall accuracy varying with the sample selection method is depicted in Figure 4, where the accuracy for dataset 1 using MLM (81%) is close to the KS algorithm (83%), which achieves the best accuracy due to the great specificity of this model. RS has the worst accuracy (79%), indicating that the performance of this method in the test set is inferior to the other algorithms that had worst fitting; thus, confirming that good fitting is not necessarily associated with good predictions.

**Table 2.** Sensitivity and specificity for the test set obtained by PCA-LDA varying with the sample selection method (RS: random selection; KS: Kennard-Stone; MLM: Morais-Lima-Martin) applied in datasets 1–6

Dataset	Sample selection method	Sensitivity (%)	Specificity (%)
1	RS	69	88
	KS	69	98
	MLM	74	78
2	RS	79	63
	KS	79	80
	MLM	81	80
3	RS		
	Class 1	83	87
	Class 2	79	89
	Class 3	89	100
	KS		
	Class 1	94	97
	Class 2	100	92
	Class 3	84	100
	MLM		
	Class 1	94	92
	Class 2	95	95
	Class 3	84	100
4	RS		
	Class 1	96	100
	Class 2	100	100
	Class 3	85	98
	Class 4	88	95
	KS		
	Class 1	100	100
	Class 2	100	100
	Class 3	90	98
	Class 4	88	97
	MLM		
	Class 1	100	100
	Class 2	100	100
	Class 3	95	98
	Class 4	88	99
5	RS	94	88
	KS	94	95
	MLM	94	91
6	RS	70	70
	KS	72	84
	MLM	72	89

Dataset 2 is composed of 140 spectra of normal (class 1) and 100 spectra of gliobastoma (class 2) brain tissue samples. Gliobastoma is the brain cancer type with the poorest survival rate (Gajjar *et al.*, 2012). Reference methods for detecting these types of cancer, such as immunohistochemical detection of isocitrate dehydrogenase (IDH), suffers from some limitations, especially their subjective nature (Gajjar *et al.*, 2012). The use of IR spectroscopy has the potential to aid tumour differentiation based on a non-analyst dependent, fast and non-destructive methodology. In this dataset, both tumour types are differentiated based on their IR spectrochemical signature. The pre-processed IR spectra for dataset 2 are show in Figure 2b. As before, RS, KS and MLM algorithms were applied to this dataset separating the data into training and test sets. PCA-LDA was applied as a classification method using 9 PCs (Fig. 3b), accounting to 99% of cumulative explained variance. The training performance of this model in dataset 2 is shown in Table 1, where the RS algorithm presents the best fitting (training and cross-validation accuracy of 85 and 83%, respectively). The other algorithms (KS and MLM) have the lowest fitting performance with accuracies around 80%. Nevertheless, as before, the situation is reversed in the test set, where the RS algorithm has the worst sensitivity and specificity values (Table 2). In the test set, the best sensitivity and specificity values are obtained using MLM, with a slightly superior performance than KS algorithm. The overall model accuracy also is better for MLM (Fig. 4), where the accuracy in the test set is observed at 81% using MLM, at 79% using KS and at 72% using RS. This confirms MLM to be the method of choice for this dataset.

Dataset 3 consists of spectra derived from SHE cells treated with one of three agents: B[a]P, class 1; 3-MCA, class 2; or, Anthracene, class 3. Class 1 is composed of 59 IR spectra, and both class 2 and 3 of 62 spectra. Pre-processed spectra for this dataset are shown in Figure 2c. PCA-LDA model was built using 10 PCs (99% cumulative explained variance) (Fig. 3c). The best training performance was found using RS algorithm, followed by MLM and KS, which had similar fitting (Table 1). KS and MLM algorithms exhibit similar performance in the test set, with sensitivities and specificities for class 1 and 2 > 90%. For class 3, both algorithms show 100% specificity and 84% sensitivity. On the other hand, the RS algorithm presents a slightly better sensitivity for class 3 (89%), but lower sensitivity and specificities for the other classes (<90%). Accuracy in the test set was found to be superior for KS (93%), followed by MLM (91%) and RS (84%) (Fig. 4). Similarly to dataset 1, KS has a slightly better performance than MLM; however, the figures of merit for MLM are more well-balanced, where extreme situations in KS (100% sensitivity or specificity) are not found, but more coherent values between these two metrics (i.e. sensitivity and specificity values closer to each other).

Dataset 4 is composed of control and typical virus-infected blood samples. Class 1 contains 90 IR spectra of control samples; class 2 contains 88 spectra of blood from patients with Dengue; class 3 contains 66 spectra of blood from patients with the Zika virus; and, class 4 contains 26 spectra of blood from patients with Chikungunya. These viruses are transmitted by mosquitos of genus Aedes, having many chemical similarities (e.g. Dengue and Zika are from the same family, Flaviviridae), in particular in their surface proteins (Santos *et al.*, 2018). Fast clinical diagnosis using reference methodologies is difficult; however, IR spectroscopy can be used as an alternative tool for viral infection differentiation (Santos *et al.*, 2018). Pre-processed spectra for dataset 4 are shown in Figure 2d. PCA-LDA model was built using 6 PCs (Fig. 3d), accounting for 97% of cumulative explained variance using RS and MLM sample selection methods, and 96% using KS sample selection method. RS and MLM exhibit similar fitting performance, with accuracies >90% in the training set. KS shows a slightly lower training performance with an accuracy of 90% in the training set (Table 1). In the test set, MLM algorithm shows the best sensitivity and specificity values (Table 2), followed by KS and RS. The overall accuracy in the test set also follows this trend, where the MLM algorithm has an accuracy of 98%, followed by KS (96%) and RS (94%) (Fig. 4).

Both datasets 5 and 6 are for diagnosis of ovarian cancer based respectively on the Raman and SERS spectra of blood plasma. These techniques have great potential towards liquid biopsy diagnosis of ovarian cancer in a minimally-invasive, rapid and objective fashion (Paraskevaidi *et al.*, 2018). Both datasets contain 2 classes, where dataset 5 is divided into 162 Raman spectra for class 1 (healthy controls) and 189 Raman spectra for class 2 (ovarian cancer); and dataset 6 is divided into 133 SERS spectra for class 1 (healthy controls) and 189 SERS spectra for class 2 (ovarian cancer). These spectra are shown in Figure 2e and f, respectively. Model construction was performed with PCA-LDA using 14 PCs (Fig. 3e and f, respectively), which accounted to 98% of cumulative variance in dataset 5 and 94% of cumulative variance in dataset 6. Training performance was superior using RS in dataset 5 and MLM in dataset 6 (Table 1), while for prediction of the external test set, the MLM algorithm showed similar classification performance in comparison with KS for dataset 5 and the best performance amongst all three algorithms in dataset 6 (Table 2 and Fig. 4), where the test accuracy for the MLM algorithm was equal to 92% in dataset 5 and 82% in dataset 6, in comparison with 94% (dataset 5) and 79% (dataset 6) using the KS algorithm and 91% (dataset 5) and 70% (dataset 6) using the RS algorithm.

**Fig. 5.**
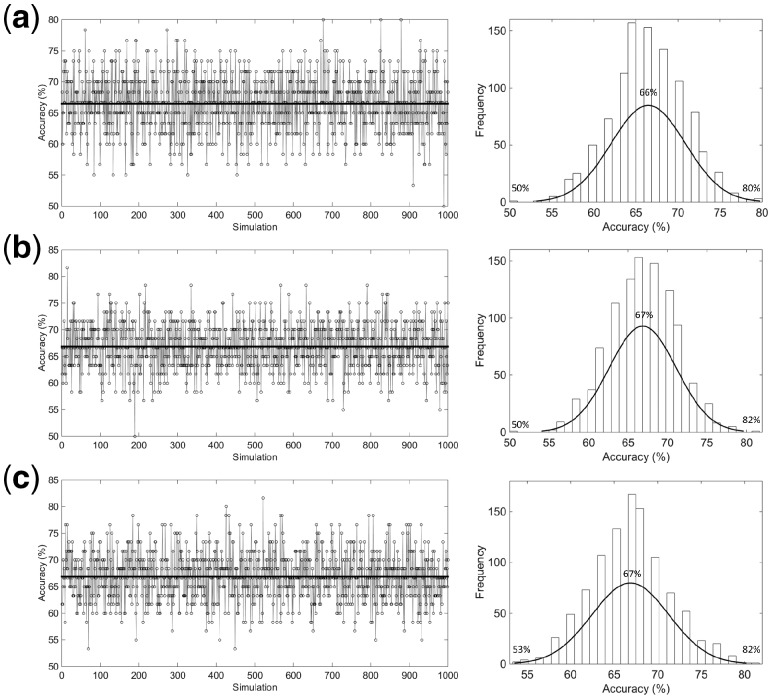
PCA-LDA accuracy distribution and histogram for 1000 simulations using normally distributed randomly data, where (**a**) RS, (**b**) KS and (**c**) MLM algorithm

Finally, 1000 simulations using a normally distributed randomly data were performed in order to compare the performance of the RS, KS and MLM algorithms in a more robust way. As depicted in Figure 5, the MLM algorithm achieved the best classification performance in terms of accuracy among all algorithms tested, with an average accuracy of 67% in the range between 53 and 82%. RS algorithm achieved the worst accuracy values, with an average of 66% and range 50–80%, while KS achieved an accuracy value similar to MLM (67%), but with a poorer lower-limit, where accuracies ranged between 50 and 82%. In addition, the histogram profiles in Figure 5 show that amongst all 1000 simulations, MLM algorithm achieved the highest frequency peak (>150 times) above the average accuracy of 67%, while for RS and KS algorithms the highest frequency peak is below the average accuracy of 67%.

These findings confirm the hypothesis that our new MLM algorithm based on a random-mutation KS algorithm approach presents a better overall performance than using RS or KS algorithms independently, especially due to the well-balanced sensitivity and specificity values in the prediction set for real-world samples. The fact that RS individually achieved good fitting but a lower predictive performance indicates that this algorithm might not include a representative variance in the training model. This reinforces the hypothesis that not necessarily an algorithm with good fitting, as demonstrated using RS, will generate good predictive results towards external samples.
